# 3-D shape reconstruction of non-uniform reflectance surface based on pixel intensity, pixel color and camera exposure time adaptive adjustment

**DOI:** 10.1038/s41598-021-83779-9

**Published:** 2021-02-25

**Authors:** Jianhua Wang, Yanxi Yang, Yuguo Zhou

**Affiliations:** 1 School of Information and Control Engineering, Qingdao University of Technology, Qingdao, 266520 China; 2 School of Automation and Information Engineering, Xi’an University of Technology, Xi’an, 710048 China

**Keywords:** Engineering, Optics and photonics, Physics

## Abstract

High dynamic range 3-D shape measurement is a challenge. In this work, we propose a novel method to solve the 3-D shape reconstruction of high-reflection and colored surfaces. First, we propose a method to establish a fast pixel-level mapping between the projected image and the captured image. Secondly, we propose a color texture extraction method using a black-and-white (B/W) camera and a pixel-level projection color adjustment method. Third, we give an optimal projection fringe modulation/background intensity ratio. Fourth, we propose a method for estimating the reflectivity of the object surface and ambient light interference, and a method for adjusting the projection intensity at the pixel level and a method for estimating the optimal exposure time. Experiments show that, compared with the existing methods, the proposed method not only can obtain high-quality captured images, but also has higher measurement efficiency and wider application range.

## Introduction

Fringe projection profilometry (FPP) is widely used in 3-D shape reconstruction, such as reverse engineering (RE), product quality control, medical diagnosis, documentation of cultural artifacts and animation production. With the advantages of high precision, high speed, full field and non-contact, this technique has become the mainstream method in non-contact 3-D shape measurement. The reconstruction accuracy of FPP depends on the captured image quality. It is difficult to obtain high-quality images on the surface of colored objects or locally highly reflective objects, which brings great challenges to 3-D shape reconstruction of the objects with non uniform reflectivity^1–5^. Regarding the above problems, many approaches have been proposed, and these methods are generally divided into the following four categories.

- Methods based on multiple exposures. Zhang et al.^6^ proposed a high dynamic range scanning technique based on a three-step phase shift method. By changing the aperture or exposure time, a series of fringe patterns with different brightness can be captured. Subsequently, the brightest but unsaturated pixels are extracted to form the final fringe pattern, which is used for phase calculation. Ekstrand et al.^7^ proposed a technique that can automatically predict the exposure time based on the reflectivity of the object surface. This technology reduces human intervention and improves the intelligence of the 3-D shape measurement system. However, choosing a single exposure time does not always adapt to the measured surface with a wide range of reflectance changes. In addition to the method of using multiple exposure times, Liu et al.^8^ also regarded the dual-camera structured light system as a two-camera-projector monocular structured light system to obtain 3-D data from different perspectives to supplement the problems caused by highlights or too dark. Jiang et al.^9^ proposed a method combining bright and dark fringe projection with multiple exposures. This method reduces the influence of ambient light, improves the signal-to-noise ratio and the dynamic range of the measurement. However, since the modulation intensity of each pixel requires a square root calculation, the amount of calculation is too large. In addition, this method proposes a set of methods for automatically selecting fringe projection brightness and exposure time parameters, but the implementation process is relatively complicated. For unknown scenes, the methods mentioned above cannot directly determine the required number of exposures and the time of each exposure at the beginning of the measurement. Zhong et al.^10^ also proposed an enhanced phase measurement profilometry, which selects an optimal exposure time to adapt to a wide range of surface reflectance changes. In the phase shift process, the exposure time should be as large as possible while ensuring that the image in the strong reflection area will not be saturated. However, this method using a single exposure time is difficult to improve the signal-to-noise ratio of the stripe pattern in the weakly reflective area. Wang et al.^11^ proposed an estimation algorithm for the exposure time interval. At the same time, in view of the requirements of DLP high-speed projection of binarized fringe patterns, an estimation method for the best four exposure times was introduced. The above method avoids redundant and useless multiple exposures, and can fuse the 3-D surfaces of four exposures, thereby obtaining a good 3-D shape of a non-uniform reflective surface.
- Polarizer-based method. For non-conductor materials, the reflected light after specular reflection is polarized, while the reflected light after diffuse reflection is not. Based on this fact, Nayar et al.^12^ installed a polarizer in front of the camera and combined the color information to separate the diffuse and specular reflection areas of the image. Salahieh et al.^13^ proposed a multi-polarization fringe projection system, which eliminates the image saturation point and enhances the fringe contrast by selecting an appropriate polarization channel. The projected fringe is polarized before incident on the object to be measured, and is captured by the camera after reflection. The camera is equipped with a pixel-level polarizer array with 4 states.
- Method based on two-color reflection model. The theoretical basis for removing highlights based on color information is the two-color reflection model proposed by Shaffer^14^. All color-based specular reflection separation methods (removing highlights) are based on the two-color reflection model. Tan et al.^15^ used the information of the surrounding area of the highlight to fill the highlight area with complementary colors. Shen et al.^16^ proposed a simple and effective method for separating reflection components in color images. This method is based on the error analysis of chromaticity. It selects the appropriate body chromaticity for each pixel, and uses the least square method to separate the diffuse reflection component and specular reflection component in the two-color reflection model. It does not require image segmentation or even interoperation between adjacent pixels to remove image highlights. Park and Lee^17^ proposed a highlight image restoration method based on color projection. It uses two images with different exposure times to easily find the highlight area of the image. Benveniste et al.^18,19^ proposed to use color invariants to solve the problem of scanning bright surfaces under different ambient lighting, and to remove the effects of highlights. They introduced a new color invariant to detect red fringes, green fringes, and highlights, so the fringes can be stably extracted from the captured images.
- Methods based on the brightness adjustment of the projected fringe pattern. Kofman et al.^20,21^ found that when measured in an uncontrollable environment, changing ambient lighting will also cause the camera to saturate. They proposed a method of reducing the maximum input gray value (MIGL) to adapt to changing ambient lighting. However, for low-reflectivity surfaces, reducing the maximum input gray value will reduce the signal-to-noise ratio of the image, so it is necessary to strike a balance between image saturation and image signal-to-noise ratio. Subsequently, Kofman et al.^22,23^ proposed to project a series of fringe patterns with a decreasing maximum input gray value, and at the same time, select pixels with the largest gray value and unsaturated pixels in the phase shift image pixel by pixel to synthesize the phase shift image and use it for phase calculation. This method has a high signal-to-noise ratio for low reflectivity surface measurement, and at the same time, it can avoid image saturation for high reflectivity surface measurement, so it can obtain higher measurement accuracy. Babaie et al.^24^ proposed a new method to improve the dynamic range of the fringe projection system. Based on the fringe image captured by the current camera, the image coordinates are first mapped to the projector image coordinate system, and then the pixels with saturated gray values are multiplied by masked image to reduce the brightness of the projection. The darker pixels of the fringe image are multiplied by masked image to increase the brightness until the camera can capture the fringe with high dynamic range. However, since the position of the calibration plate is not consistent with the position of the measured object, the transformation matrix and translation vector obtained by this method are not accurate enough. Zhang et al.^25^ used monochrome black and white fringe patterns to measure objects with different reflectances, thereby improving robustness. They used an adaptive intensity mask to dynamically adjust the pattern intensity to prevent overexposure of bright areas. This Coded mask is derived from point spread function and camera response function. Compared with traditional methods, this method usually requires three iterations to quickly find the point spread Function. The point spread function is based on the homography matrix between the camera image plane and the projector image plane, which can be obtained by the advanced measurement platform calibration. However, it is difficult to ensure the position of the measured object during measurement and the position of the calibration board is completely consistent, so the coordinates after the homography matrix mapping are not very accurate.

In this paper, we first proposed a novel pixel matching method, which established the pixel matching between the projected image and the captured image. Then, we proposed a B/W camera-based color information extraction method, an object surface reflectance and ambient light interference estimation method. Finally, the pixel color, pixel intensity and exposure time can be adaptively adjusted according to the color information and reflectivity of the measured objects.

## Principle of the proposed method

### Establish the pixel relationship between the captured and projected images

**Figure 1:**
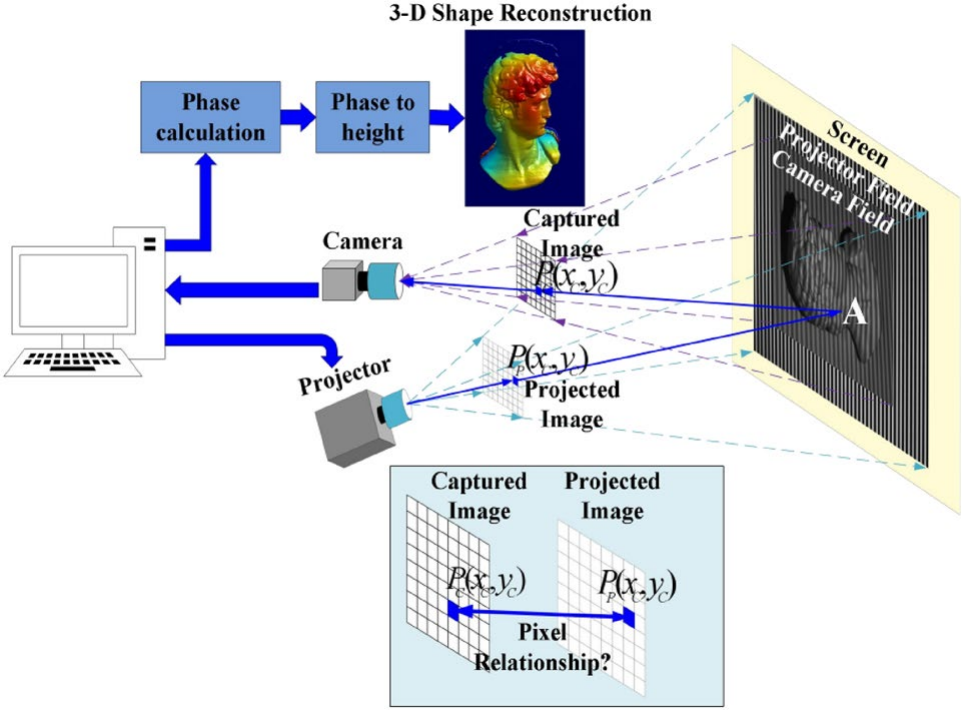
Camera and projector field of view.

As shown in Fig. 1, the pixel ${P_C}({x_C},{y_C})$ in the camera image coordinate system correspondence to the pixel ${P_P}({x_P},{y_P})$ in the projection image coordinate system need to be mapped. Due to the height changes of different measured objects, the uncertainty of the field of view of projection and the field of view of the camera, the spatial position relationship between ${P_C}({x_C},{y_C})$ and ${P_P}({x_P},{y_P})$ cannot be described by a definite rotation and translation matrix. Therefore, it is necessary to efficiently establish this mapping relationship before each 3-D measurement^26^. In this work, we proposed a new method to solve this problem, the detailed process is as follows.

**Figure 2:**
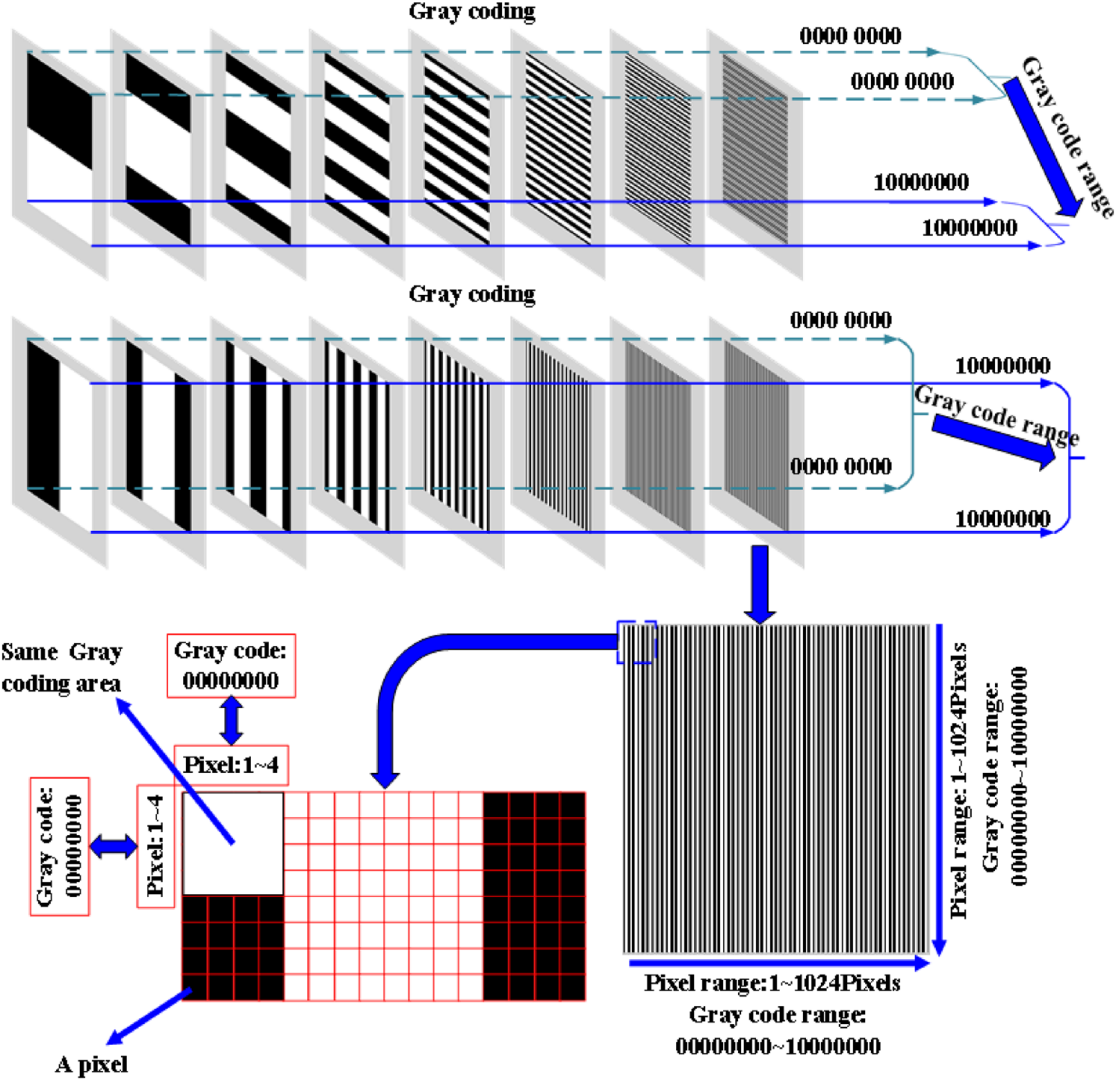
Rough mapping based on Gray coding method.

**Step 1. Rough mapping method.** In this work, we use DLP Light Craft 4500 for projection. The size of the projected image is 912 × 1140 pixels, and the number of gray coding bits is 8. Therefore, the coarse coding divides the projected image into 256 × 256. Since round(912 pixels/256) = 4 pixels and round(1140 pixels/256) = 4 pixels, 4*4 matrix pixels will correspond to the same code. For example, the 4*4 area shown in Fig. 2 has the same code. This step only realizes the coarse pixel mapping between the projection and the captured image.

**Step 2. Fine mapping method.** The Gray coded image moves cyclically in the row and column directions^27–31^, and the number of moves is round(912 pixels/256) = 4 pixels and round(1140 pixels/256) = 4 pixels. We take the gray coded image circularly moving along the row direction as an example, the details of the algorithm are described as follows:

**Step 2.1.** We cyclically move the Gray coded image by 1 bit in the row direction, and then decode the captured image. Assume that *M* and *N* are the row and column decoding results of the coarse coding of the pixel. If the decoding result changes, the correct coding value of the pixel is 4*(*M*-1) + 1. If the decoding result does not change, we will perform the step 2.2.

**Step 2.2.** We cyclically move the Gray coded image by 1 bit in the row direction again. If the decoding result changes, the correct code value of the pixel is 4*(*M*-1) + 2. If the decoding result does not change, we will perform the step 2.3.

**Step 2.3.** We cyclically move the Gray coded image by 1 bit in the row direction again. If the decoding result changes, the correct code value of the pixel is 4*(*M*-1) + 3. If the decoding result does not change, we will perform the step 2.4.

**Step 2.4.** We cyclically move the Gray coded image by 1 bit in the row direction again. If the decoding result changes, the correct code value of the pixel is 4*(M-1) + 4. Otherwise, we define it as an outlier.

**Step 3. Outlier detection, elimination and replacement.** Due to the errors of round(912 pixels/256) = 4 pixels and round(1140 pixels/256) = 4 pixels, as well as light scattering and ambient lighting, there may be local errors in the encoding results. The details of the proposed method are as follows:

**Step 3.1.** Construction of row and column coded value matrix. The row coding result and the column coding result are extracted from the coding result of each pixel of the captured image to form two matrices **M**_1_ and **M**_2_. The size of the two matrices is consistent with the size of the captured image.

**Step 3.2.** Elimination of outliers. We use the outlier elimination method based on Grubbs criterion to convolve the matrices **M**_1_ and **M**_2_, and take the convolution window size of 5*5 pixels.

**Step 3.3.** Outliers are filled. Outliers are filled. We search the four neighborhoods of outliers, calculate the difference between the upper and lower codes and the difference between the left and right codes, find the two codes with the smaller difference and average them to fill in the outliers.

The proposed method needs to project 12 × 4 images (8 Gy coded images and four sets of pixel displacement) before measurement to establish the pixel mapping between the projection and the captured image. In order to increase the speed of this step, we use high-speed projection technology. The capture speed can be very high, such as the Daheng image camera MER-050-560U3M at a resolution of 800*600 pixels up to 560 fps. However, the refresh rate of the projector is limited, and its refresh rate is related to the bit depth of the projected image. Such as DLP LightCraft 4500 from Texas Instruments, the maximum refresh rate for projecting 8-bit images is 120 Hz, while the refresh rate for 1-bit images is as high as 4225 Hz. Since the Gray coded image is a binary image, high-speed projection can be realized. In this work, we choose the maximum frame rate of 560fps according to the maximum frame rate of the camera MER-050-560U3M. Therefore, it only takes 0.086 s (86 ms) to project 48 images.

DLP Lightcraft 4500 requires that the projected image must be a 24-bit image, and the number of 24-bit images cannot exceed two, that is, no more than 48 bits in total. Therefore, it is necessary to synthesize 48 1-bit Gray coded images into two 24-bit fringe images, where the first 24-bit image is obtained by formula (1), and the second is omitted here.

1 $$\begin{aligned}\left\{ {\begin{array}{*{20}{ll}} {{f_{{\text{red}}}}(x,y) = {2^0}\left[ {{f_{{\text{binary\_}}1}}(x,y)} \right] + {2^1}\left[ {{f_{{\text{binary\_}}2}}(x,y)} \right] + {2^2}\left[ {{f_{{\text{binary\_}}3}}(x,y)} \right] + {2^3}\left[ {{f_{{\text{binary\_4}}}}(x,y)} \right] }\vspace{5pt}\\\quad{+ {2^4}\left[ {{f_{{\text{binary\_5}}}}(x,y)} \right] + {2^5}\left[ {{f_{{\text{binary\_6}}}}(x,y)} \right] + {2^6}\left[ {{f_{{\text{binary\_7}}}}(x,y)} \right] + {2^7}\left[ {{f_{{\text{binary\_8}}}}(x,y)} \right]} \vspace{5pt}\\ {{f_{{\text{green}}}}(x,y) = {2^0}\left[ {{f_{{\text{binary\_}}9}}(x,y)} \right] + {2^1}\left[ {{f_{{\text{binary\_}}1{\text{0}}}}(x,y)} \right] + {2^2}\left[ {{f_{{\text{binary\_}}1{\text{1}}}}(x,y)} \right] + {2^3}\left[ {{f_{{\text{binary\_12}}}}(x,y)} \right] }\vspace{5pt}\\\quad{+ {2^4}\left[ {{f_{{\text{binary\_13}}}}(x,y)} \right] + {2^5}\left[ {{f_{{\text{binary\_14}}}}(x,y)} \right] + {2^6}\left[ {{f_{{\text{binary\_15}}}}(x,y)} \right] + {2^7}\left[ {{f_{{\text{binary\_16}}}}(x,y)} \right]} \vspace{5pt}\\ {{f_{{\text{blue}}}}(x,y) = {2^0}\left[ {{f_{{\text{binary\_}}17}}(x,y)} \right] + {2^1}\left[ {{f_{{\text{binary\_}}1{\text{8}}}}(x,y)} \right] + {2^2}\left[ {{f_{{\text{binary\_}}1{\text{9}}}}(x,y)} \right] + {2^3}\left[ {{f_{{\text{binary\_20}}}}(x,y)} \right]}\vspace{5pt}\\\quad{ + {2^4}\left[ {{f_{{\text{binary\_21}}}}(x,y)} \right] + {2^5}\left[ {{f_{{\text{binary\_22}}}}(x,y)} \right] + {2^6}\left[ {{f_{{\text{binary\_23}}}}(x,y)} \right] + {2^7}\left[ {{f_{{\text{binary\_24}}}}(x,y)} \right]} \end{array}} \right.\end{aligned}$$

where *f*_red_, *f*_green_ and *f*_blue_ are the red, green and blue components of the 24-bit image, *f*_binary_1_, *f*_binary_2_, –, *f*_binary_24_ are 24 Gy binary images.

### Use the B/W camera to capture the color texture

Colorful objects can be seen everywhere. When a color camera is adopted to capture the fringe pattern on the surface of a color object, there is a problem of information crosstalk between the information components of each channel. In this work, we use B/W camera to capture fringe patterns, and propose a simple and easy method for color texture extraction.

**Figure 3:**
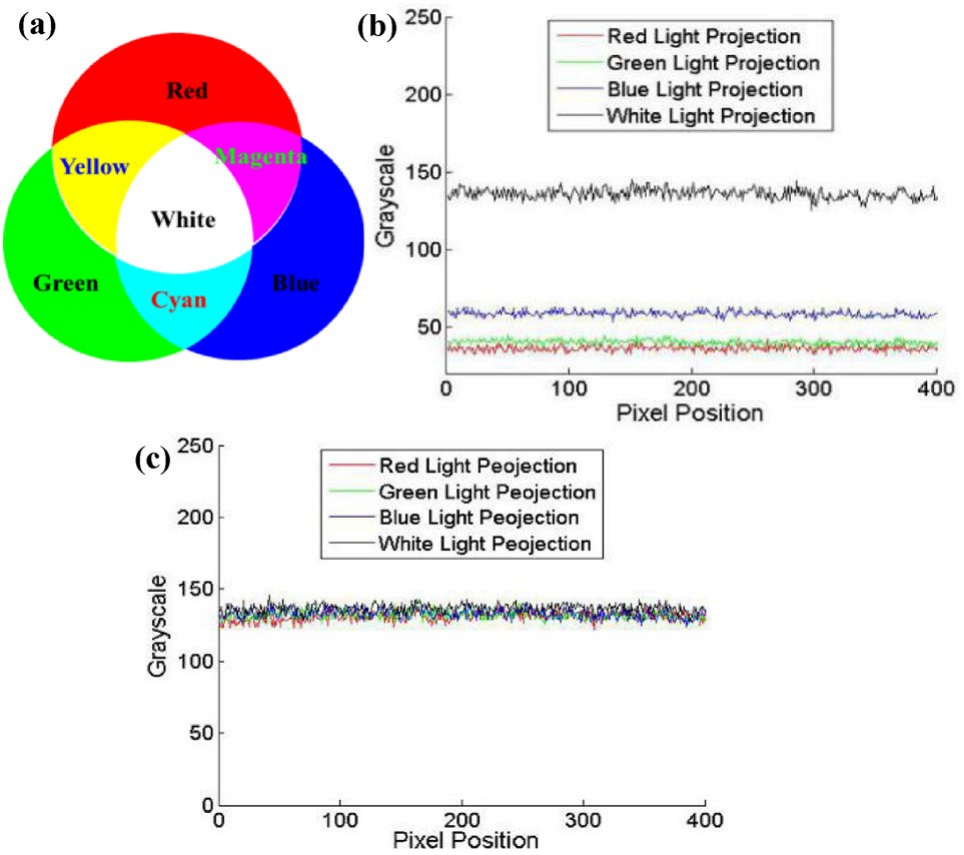
The principle of color texture extraction using B/W camera. (**a**) Additive method of three primary colors. (**b**) The intensity of the captured images employing white, red, green and blue light projection. (**c**) The intensity of the captured images after the exposure time adjustment.

All the colors can be synthesized from three primary colors (Red, Green and Blue, i.e. RGB) in different proportion. Additive method of three primary colors is shown in Fig. 3a. We project RGB light onto the whiteboard and use a B/W camera to capture the images. The color of whiteboard can be synthesized from the captured images.

2 $${I^{Color}}(x,y) = cat(3,{I^{Red}}(x,y),{I^{Green}}(x,y),{I^{Blue}}(x,y)),$$

where *I*^*Color*^ represents the color of the measured object, *I*^*Red*^*, I*^*Greeb*^ and *I*^*Blue*^ represent the grayscale captured by a B/W camera when RGB light is projected, *cat* is a operator to concatenate arrays along specified dimension.

Figure 3b show the intensity of a line of pixels of the captured images. It can be seen from Fig. 3b that the intensity of the captured images is different. However, for the whiteboard, the intensity of the captured images should be equal to get white texture correctly, so we must make necessary adjustment to ensure that the grayscale of the three images is close to each other.

The intensity of the projected image is constant, the intensity of the captured image is determined by the camera sensitivity. The camera sensitivity can be expressed as:

3 $${C_S} = f({C_A},{C_T}),$$

where *C*_*S*_ represents the camera sensitivity, *C*_*A*_ represents the aperture of camera lens, *C*_*T*_ is the camera exposure time.

The projected fringe pattern employing phase-shifting (PS) algorithm can be expressed as:

4 $$I_P^n({x_P},{y_P}) = A({x_P},{y_P}) + B({x_P},{y_P}){\kern 1pt} \cos [\phi ({x_P},{y_P}) + 2\pi (n - 1/N],$$

where $I_P^n$ represents the intensity of the *n*th projected fringe pattern, (*x*_*P*_, *y*_*P*_) is the coordinate of a pixel on the projected image, *A* is the average intensity of the projected fringe pattern, *B* is the modulation intensity of the projected fringe pattern, *n* represents the *n*th phase shifting, *N* represents the total number of phase shifting.

The captured fringe pattern can be expressed as:

5 $$I_C^n({x_C},{y_C}) = {C_S}\left[ {r({x_C},{y_C})I_P^n({x_P},{y_P}) + {A_L}({x_C},{y_C})} \right],$$

where $I_C^n$ represents the intensity of the *n*th captured fringe pattern, *r* represents the reflectivity of the object surface, *A*_*L*_ represents the ambient light.

Parameters *r* and $I_P^n$ are constant. Since adjusting the aperture of camera lens is not automatic and quantitative, adjusting the camera exposure time is adopted in this work. The captured fringe pattern can be rewritten as:

6 $$I_C^n({x_C},{y_C}) = {C_T}\left[ {r({x_C},{y_C})I_P^n({x_P},{y_P}) + {A_L}({x_C},{y_C})} \right],$$

where *T*^*C*^ is the camera exposure time.

**Figure 4:**
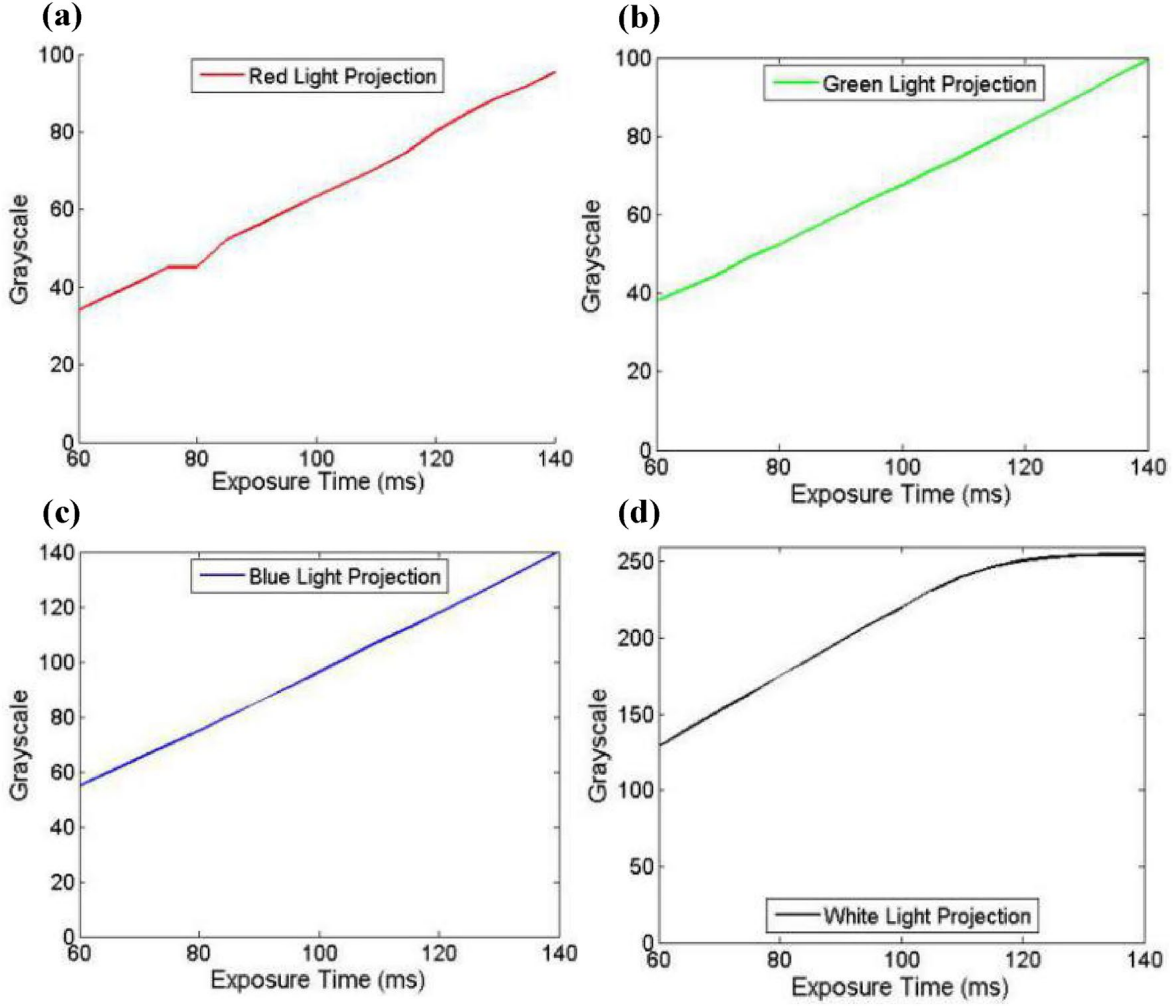
Characteristic of the camera at different exposure time. (**a**) Red light projection. (**b**) Green light projection. (**c**) Blue light projection. (**d**) White light projection.

Figures 4 show the camera characteristics at different exposure time. Gray value is obtained by calculating the average value of the whole image. It can be seen that the camera characteristic is approximately first-order linear on the premise that the exposure time is not too high and the captured image is not overexposed. In order to eliminate the non-linear segment and overexposure segment, we choose the exposure time interval of 60–100 for fitting. This is reasonable because the fringe images captured in 3-D measurement cannot be overexposed as well.

7 $$\left\{ {\begin{array}{*{20}{l}} {{I^R} = 2.2849{C_T}{ - 7}{\text{.8197}}} \\ {{I^G} = 0.7739{C_T}{ - 9}{\text{.3427}}} \\ {{I^B} = 1.0518{C_T}{ - 8}{\text{.5738}}} \\ {{I^W} = 0.712{C_T}{ - 6}{\text{.9571}}} \end{array}} \right.,$$

where *I*^*R*^*, I*^*G*^*, I*^*B*^ and *I*^*W*^ represent the average gray value when RGB and white light are projected.

**Figure 5:**
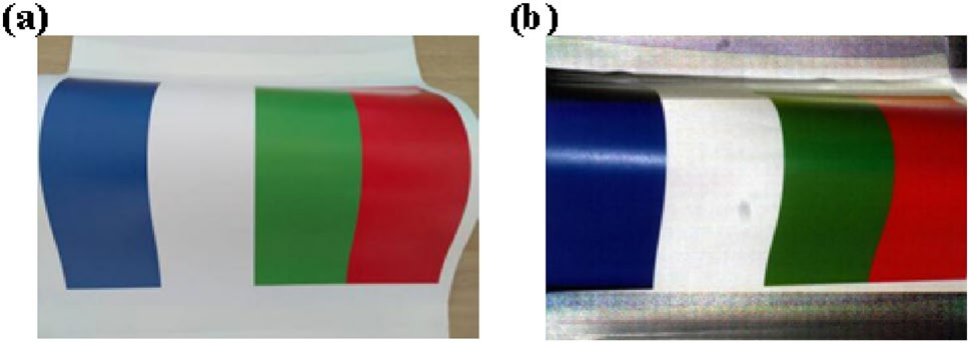
The color texture extraction. (**a**) The colorful object. (**b**) The extracted color texture.

After adjusting the exposure time, the grayscale of the whiteboard is consistent, as shown in Fig. 3c. For example, we take an object that has four colors on the surface as the measured object, as shown in Fig. 5a. We can obtain the correct color by synthesizing the captured images according to Eq. (1). The extracted color texture is shown in Fig. 5b.

### Pixel-level color adjustment of the projected image

Since different color on the object surface can absorb different wavelength of the projected light, the contrast and brightness of the captured images are not uniform based on monochromatic light projection, which will reduce the measurement accuracy. 3D shape reconstruction of colorful objects is still a challenge.

**Figure 6:**
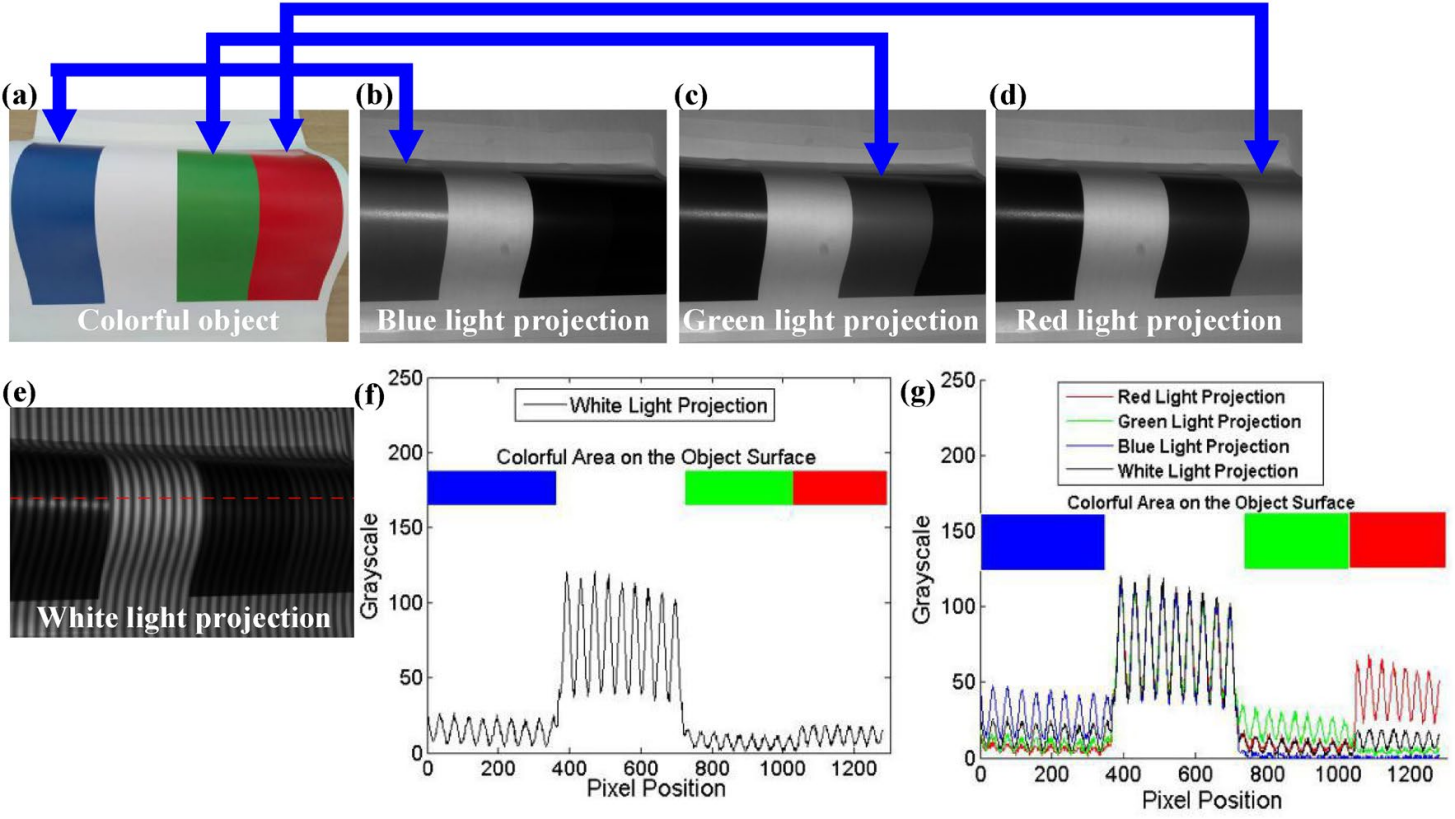
Pixel-level color. (**a**) Colorful object. (**b**) The captured image by a B/W camera based on blue light projection. (**c**) The captured image by a B/W camera based on green light projection. (**d**) The captured image by a B/W camera based on red light projection. (**e**) The captured fringe pattern by a B/W camera based on white light projection. (**f**) The grayscale of the red dotted line in (**e**). (**g**) The grayscale of the red dotted line in (**e**) based on different light projection.

For example, we take an object shown in Fig. 6a as the measured object. Figure 6b–d are the captured images by a B/W camera based on blue, green and red light projection. It can be seen that no matter what color light is projected to colorful objects, the intensity of the captured image on the white surface is relatively high. When we project blue light, the brightness of the captured image on the blue surface is relatively high, however, the brightness of the captured image on the surface of other colors is relatively low. In addition, when we project green or red light, the brightness on the green or red surface is relatively high.

We further discuss the contrast of the captured images. First, we project the white fringe pattern, and the captured image is shown in Fig. 6e. We extract a row of pixels at the red dotted line in Fig. 6e to get the intensity and contrast shown in Fig. 6f. It can be seen that the intensity and contrast of the white surface is higher, but the intensity and contrast of other colored surfaces are lower. We project the blue, green and red fringe patterns, and then extract three rows of pixels at the red dashed line in Fig. 6e, the results are shown in Fig. 6g. Obviously, the brightness and contrast on the blue surface based on blue light projection are higher than those based on other color light projections. Other results are similar. Therefore, for 3-D shape measurement of colorful objects, we can obtain the surface color texture of the object according to the method proposed in Section B, and then adjust the color of each pixel in the projected image according to the method proposed in Section A. Pixel-level color adjustment of the projected image can be expressed as:

8 $${C_P}({x_P},{y_P}) = cat(3,I_C^{Red}({x_C},{y_C}),I_C^{Green}({x_C},{y_C}),I_C^{Blue}({x_C},{y_C}))$$

where ${C_P}({x_P},{y_P})$ represents the color of the pixel $({x_P},{y_P})$ on the projected image, $I_C^{Red}({x_C},{y_C})$*,* $I_C^{Green}({x_C},{y_C})$ and $I_C^{Blue}({x_C},{y_C})$ represent the grayscale of the pixel $({x_C},{y_C})$ on the captured image when RGB light is projected.

Through the method described in Section A, the pixel $({x_P},{y_P})$ on the projected image corresponding to the pixel $({x_C},{y_C})$ on the captured image can be obtained.

### Optimal projection fringe modulation/background intensity ratio

According to formula (6), the captured fringe pattern is related to the projected fringe pattern, camera exposure time, and ambient light. The average intensity of the captured fringe pattern is

9 $$a_C^n({x_C},{y_C}) = {C_T}\left[ {r({x_C},{y_C})A({x_C},{y_C}) + {A_L}({x_C},{y_C})} \right]$$

The intensity modulation of the captured fringe pattern is

10 $$b_C^n({x_C},{y_C}) = {C_T}r({x_C},{y_C})B({x_C},{y_C})$$

The data quality of the captured fringe pattern can be expressed as

11 $${\gamma^n}({x_C},{y_C}) = \frac{{b_C^n({x_C},{y_C})}}{{a_C^n({x_C},{y_C})}} = \frac{{r({x_C},{y_C})B({x_C},{y_C})}}{{r({x_C},{y_C})A({x_C},{y_C}) + {A_L}({x_C},{y_C})}}$$

The larger the *γ*, the higher the SNR of the captured fringe pattern, and the better the phase calculation. *γ* ∈ (0, 1], so we must make *γ* close to 1 to achieve high-quality measurement. We can obtain the ideal ratio of the projection fringe modulation/background intensity.

12 $$B({x_C},{y_C}) = A({x_C},{y_C}) + \frac{{{A_L}({x_C},{y_C})}}{{r({x_C},{y_C})}}$$

### Determination of the environmental intensity and the reflectance of the surface with a wide range of reflectance changes

In this work, we project two additional images to estimate *r*(*x*_*C*_, *y*_*C*_) and *A*_*L*_(*x*_*C*_, *y*_*C*_) in formula (6). The grayscale of the first and second projection images is 0 and 100, respectively. In order to ensure that the captured fringe patterns are not overexposed, we choose a smaller camera exposure time. We project and capture them, *r*(*x*_*C*_, *y*_*C*_) and *A*_*L*_(*x*_*C*_, *y*_*C*_) can be calculated according to Eq. (13).

13 $$\left\{ {\begin{array}{*{20}{l}} {A_L^*({x_C},{y_C}) = \frac{{I_C^0({x_C},{y_C})}}{{C_T}}} \\ {{r^*}({x_C},{y_C}) = \frac{{I_C^{100}({x_C},{y_C})}}{{{100}{C_T}}}{ - }\frac{{{A_L}({x_C},{y_C})}}{{{100}}}} \end{array}} \right.$$

where $I_C^0$ and $I_C^{100}$ represents the captured fringe pattern when the intensity of the projected image is 0 and 100.

### Determination of the optimal projection pixel intensity in the camera image coordinate system

In formula (6), parameters ${I_P}({x_C},{y_C})$ and *C*_*T*_, that is, the projected pixel intensity in the camera image coordinate system and camera exposure time, need to be optimized. The details of the algorithm are described as follows:

**Step 1. Optimizing constraints.** In formula (6), $I_C^n$ and $I_P^n$, that is, the pixel intensity of the projected and captured images, must be in the interval 0 ~ 255. Considering the calculation error, the maximum intensity is 250. The camera exposure time of MER-050-560U3M from Daheng imaging must be in the interval 20 ~ 1000000 μs.

**Step 2. Optimizing goal.** The pixel intensity of the captured image should be as large as possible to improve the SNR of the captured fringe pattern.

**Step 3. Determination of the initial maximum C**_***T1***_**.** The pixel intensity of the projected image is all set to 250, which is substituted into the formula (6). The exposure time is substituted into the formula (6) in descending order. *C*_*T*1_ is the maximum exposure time that can make all pixels in the captured image does not exceed 250.

**Step 4. Determination of the best projection pixel intensity in the camera coordinate system.** We substitute *C*_*T*1_ into the formula (6), and make the pixel intensity of the captured image to be the maximum 250. Subsequently, we calculate the projection pixel intensity according to the formula (6). If the pixel intensity is greater than 250, it is 250. So the best projection fringe pattern, $I_{OP}^n$, is determined.

**Step 5. Determination of the final maximum C**_***TO.***_ We substitute *C*_*T*1_ and $I_{OP}^n$ into formula (6), and extract the maximum gray value *G*_*M*_ from the captured fringe pattern, then the final exposure time can be obtained by the following formula.

14 $${C_{TO}} = \frac{250}{{G_M}}{C_{T1}}$$

### High-precision phase calculation

Three-step phase-shifting (PS) method is adopted to calculate the wrapped phase.

15 $${\psi_{\text{3 - step}}}(x,y) = {\tan^{ - 1}}\left\{ {\frac{{\sqrt 3 [{I_2}(x,y) - {I_3}(x,y)]}}{{2{I_1}(x,y) - {I_2}(x,y) - {I_3}(x,y)}}} \right\}$$

where *ψ*_*3-step*_(*x, y*) represents the wrapped phase using three-step PS. Due to the application of the tan^−1^ operator, *ψ*_*3-step*_ is between − *π* and *π* when considering the sign of the real and imaginary parts^32–35^.

The real phase can be written as.

16 $$\phi (x,y) = 2\pi k + {\psi_{\text{3 - step}}}(x,y)$$

where *k* is the fringe order, the process of obtaining real phase is called phase unwrapping.

Phase unwrapping algorithms are generally divided into two categories, spacial phase unwrapping (SPU) and temporal phase unwrapping (TPU) algorithms. TPU algorithms need to project and capture fringe sequences with different frequencies, but its phase unwrapping accuracy and computation reliability are very high. At present, three pitches unwrapping algorithm (TPUA), negative exponential unwrapping algorithm (NEUA) and three pitches heterodyne unwrapping algorithm (TPHUA) are widely used. TPHUA only needs three frequency fringe sequences, while ensuring higher reliability in the unwrapping stage^36–38^.

Principle of TPHUA is as follows. Three frequencies of the projected fringe patterns are *f*1 = *S* + $\sqrt S$ + 1 (*S* is a constant), *f*2 = *S* and *f*3 = *S − *$\sqrt S$, respectively. ${\psi^{S + \sqrt S + 1}}$, ${\psi^S}$ and ${\psi^{S - \sqrt S }}$ are calculated by three-step PS. ${\psi^{\sqrt S + 1}}$ is obtained from ${\psi^{S + \sqrt S + 1}}$ and ${\psi^S}$, ${\psi^{\sqrt S }}$ is obtained from ${\psi^S}$ and ${\psi^{S - \sqrt S }}$. At last, *ψ*^1^ is calculated from ${\psi^{\sqrt S + 1}}$ and ${\psi^{\sqrt S }}$. *φ*^1^ = *ψ*^1^*,* the unwrapped phase with higher frequency can be obtained using the Formula (15).

17 $$\left\{ {\begin{array}{*{20}{l}} {{\phi^{f_i}}(x,y) = {\psi^{f_i}}(x,y) + {R^{f_i}}(x,y) \times 2\pi } \\ {{R^{f_i}}(x,y) = INT\left[ {\frac{{({f_i}/{f_{(i - 1)}}) \times {\phi^{{f_{(i - 1)}}}}(x,y){ - }{\psi^{f_i}}(x,y)}}{2\pi }} \right]} \end{array}} \right.$$

where *ψ*^*f*^ and *φ*^*f*^ represents the wrapped and unwapped phase with the frequency *f*, *INT* is a *round* operator.

## Experiments

**Figure 7:**
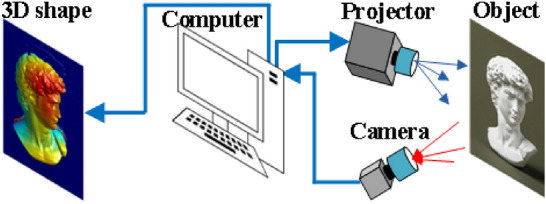
Structure of the 3-D measurement system.

The structure of the 3-D measurement system is shown in Fig. 7, including a projector, a camera and a personal computer. The computer reconstructs 3-D shape after processing the captured fringe patterns.

### Experimental results of the smooth metal objects

**Figure 8:**
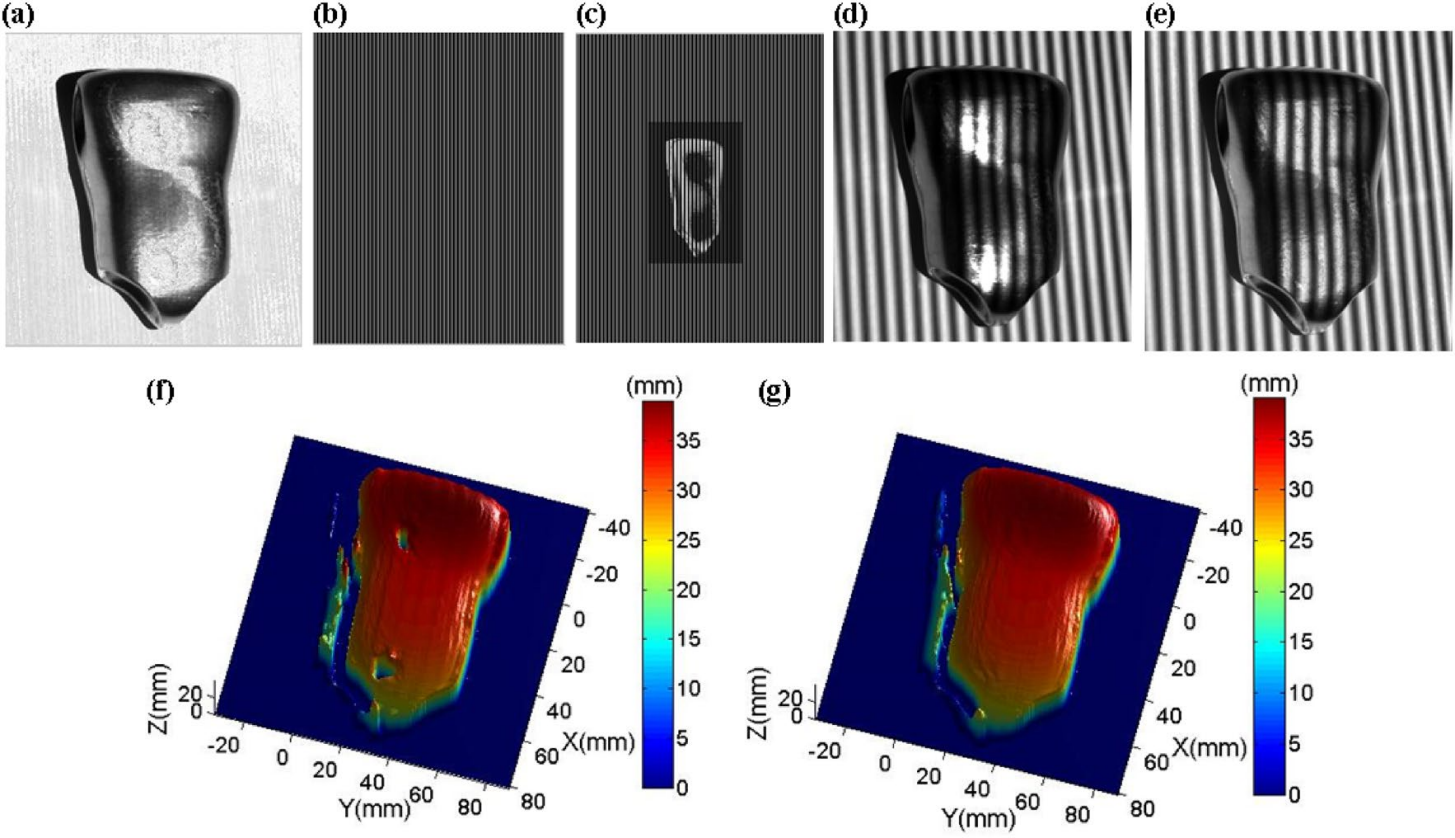
3-D shape reconstruction of a metal product 1. (**a**) A metal product. (**b**) Original projection fringe pattern. (**c**) Optimized projection fringe pattern. (**d**) Original captured fringe pattern. (**e**) Optimized captured fringe pattern. (**f**) Reconstructed 3-D surface shape using the original captured fringe patterns. (**g**) Reconstructed 3-D surface shape using the optimized captured fringe patterns.

After metal is processed at high speed on various machines, its surface is often very smooth, so the captured image has non-diffuse reflection characteristics. Figure 8a is a captured image of a metal product 1 processed at a high speed on a CNC milling machine. The original projection fringe pattern is shown in Fig. 8b. Based on the adaptive projection fringe optimization method proposed in this paper, we can get a projection fringe patter shown in Fig. 8c. It can be seen from Fig. 8c that for the different reflectivity of the measured surface, the pixel intensity on the corresponding projection image is adjusted adaptively. Since there are no colors on the surface, the adaptive projection fringe pattern is pure color. For colored surfaces, we will analyze and compare in Sect. 3.2. Figure 8d is an original captured fringe pattern. The high reflectance area of the metal surface is overexposed, and the low reflectance area is too dark. Over-exposure will cause phase calculation errors, while over-darkness will reduce image contrast. Figure 8e is a captured fringe pattern based on the adaptive fringe projection proposed in this paper. The overexposed area is significantly improved, and the pixels in the over dark area become brighter. In fact, the intensity and contrast of the over dark area are improved. We compare the 3-D reconstruction surfaces, and the results are shown in Fig. 8f,g. Using original fringe patterns, there are holes or wrinkles in the 3-D surface shape of the overexposed and over-dark areas. However, the 3-D surface shape using the optimized captured fringe patterns has been significantly improved.

To achieve the quantitative comparisons of the metal product 1, we utilize RMSE (root mean square error) to evaluate the 3-D shape precision. It can be seen from Table 1 that the measurement error of the proposed method is reduced by 50.7%.

**Table 1:** Quantitative comparison of the metal product 1 (RMSE).

Method	Without the proposed method	With the proposed method
RMSE	0.221	0.109

In addition, we employed maximum discrepancy (MD) to evaluate accuracy. MD is defined as.

18 $${\text{MD}} = \max \left\{ {\sum\nolimits_{y = {1}}^N {\sum\nolimits_{x = {1}}^M {\left[ {{h_i}(x,y) - {h_r}(x,y)} \right]} } } \right\}$$

where max is the operator to search for the maximum value, *h* is the height of the 3-D shape.

Table 2 shows the MD value of the 3-D reconstruction shape. Without the proposed method, the MD value of the reconstructed 3-D is 1.83 mm. However, the MD value of the proposed method is 0.51 mm, which can be used for industrial 3D measurement.

**Table 2:** Quantitative comparison of the metal product 1 (MD).

Method	Without the proposed method	With the proposed method
MD (mm)	1.83	0.51

In addition, regarding the measurement efficiency, the proposed method needs to add two images when calculating the adaptive projection fringe pattern. When extracting the color texture of the surface of the measured object, three images are added. Therefore, the proposed method only needs to add 5 images. However, references^6–8^ require a large number of camera exposure time adjustment or projection image intensity adjustment, and the number of additional images is very large. We compare the proposed method with references^6, 7^, the number of additional projection fringes is shown in Table 3, where *N* represents the number of fringe patterns in a set of fringe sequences.

**Table 3:** Number of additional fringe patterns.

Method	Ref.^6^	Ref.^7^	The proposed method
Number of additional fringe patterns	22*N	6*N	5

Reference^6^ uses 23 camera exposure times to get the fringe sequence, and 22 additional sets of fringe sequences are added. Reference^7^ obtains fringe sequences with different intensities by using colored light projection, intensity adjustment of the entire projected image, and camera exposure time adjustment. Compared with a set of fringe sequences, 6 sets of fringe sequences are added. However, the method proposed in this paper only needs to add 5 additional fringe patterns to get a good set of fringe patterns. It is worth noting that there is a big difference between the method in this paper and the method in reference^7^. That is, in reference^7^, the overall grayscale of the projected fringe pattern is adjusted. The method proposed in this paper is to adjust each pixel on the projected fringe pattern according to the reflectivity of the measured surface.

**Figure 9:**
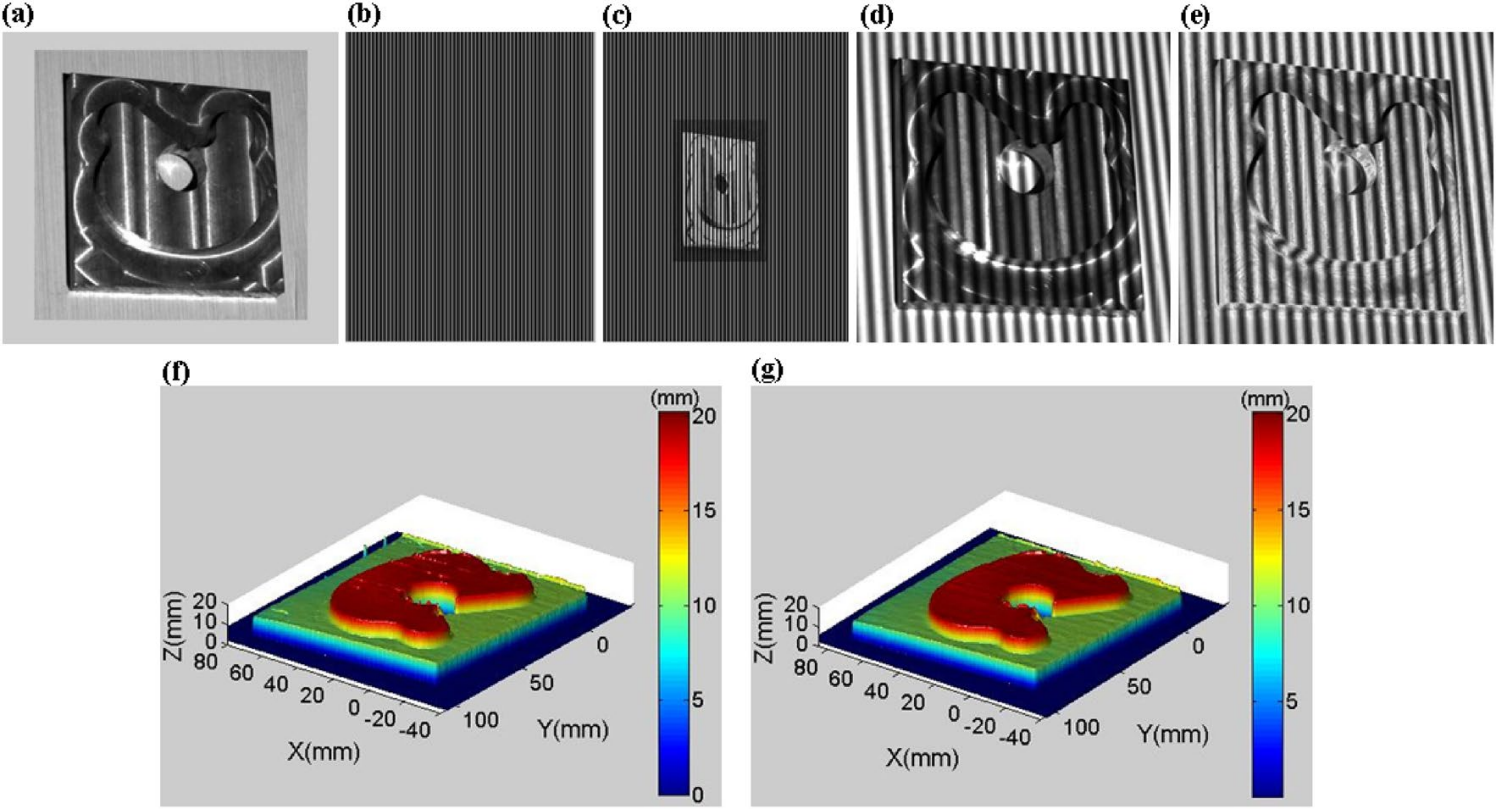
3-D shape reconstruction of another metal product 2. (**a**) Another metal product. (**b**) Original projection fringe pattern. (**c**) Optimized projection fringe pattern. (**d**) Original captured fringe pattern. (**e**) Optimized captured fringe pattern. (**f**) Reconstructed 3-D surface shape using the original captured fringe patterns. (**g**) Reconstructed 3-D surface shape using the optimized captured fringe patterns.

To further verify the effectiveness of the proposed method, we take another metal product 2 as the measured objects, as shown in Fig. 9a. Figure 9b is an original projection fringe pattern. Figure 9c is an adaptive projection fringe pattern, and its pixel intensity is adjusted adaptively according to the reflectivity of the measured object. Figure 9d is an original captured fringe pattern. Figure 9e is an optimized captured fringe pattern based on the adaptive fringe projection proposed in this paper. Figures 9f,g show the 3-D reconstruction surfaces. It can be seen that the 3-D surface shape using the proposed method is greatly improved.

For quantitative comparisons of the metal product 2, we utilize RMSE and MD to evaluate the 3-D shape precision. The comparison results are shown in Table 4. The RMSE and MD errors based on the proposed method are reduced by 59.9% and 71.3%, respectively.

**Table 4:** Quantitative comparisons of the metal product 2 (RMSE and MD).

Method	Without the proposed method	With the proposed method
RMSE	0.0681	0.0273
MD (mm)	1.57	0.45

### Experimental results of colorful objects

**Figure 10:**
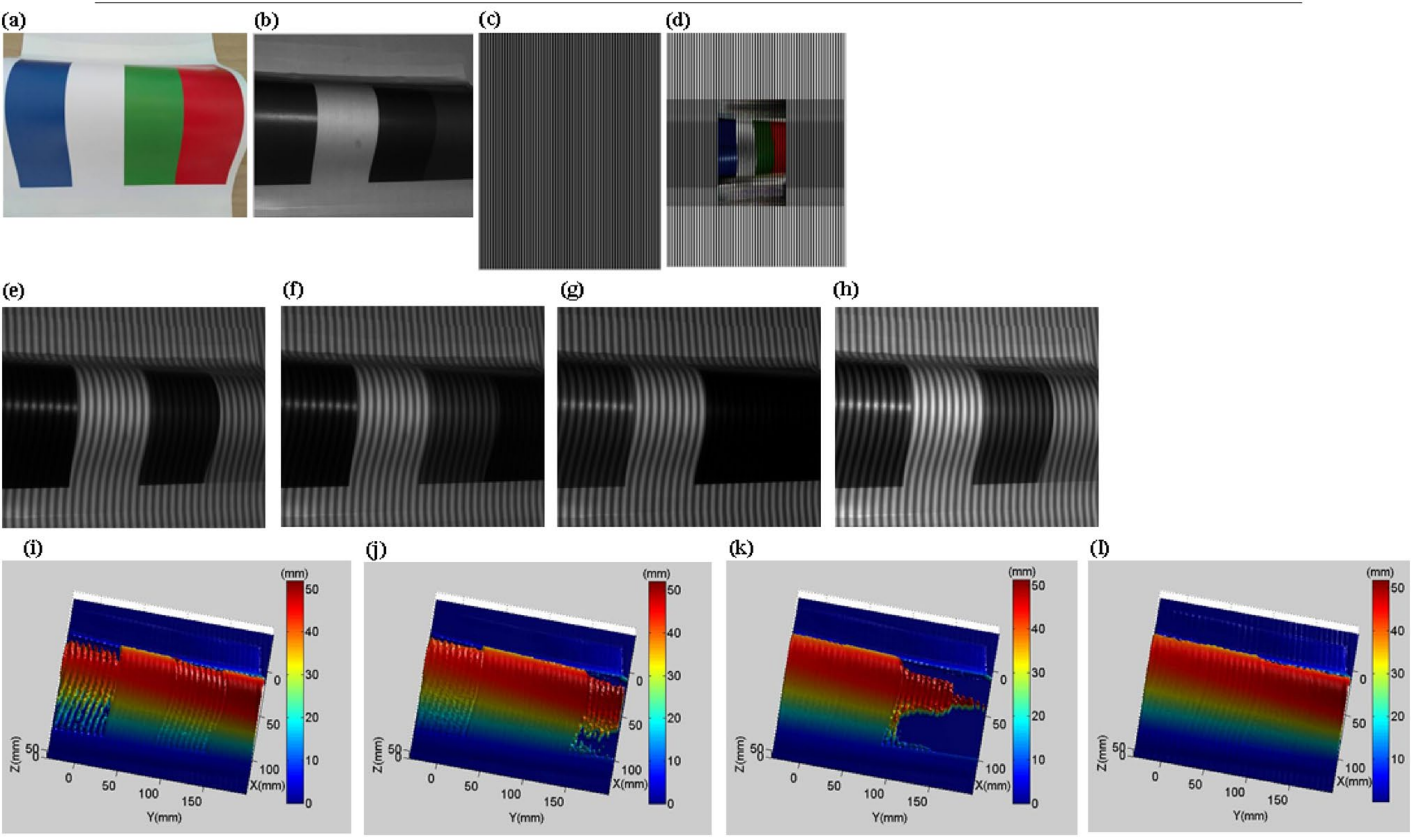
3-D shape reconstruction of a colorful object 1. (**a**) A colorful object. (**b**) A captured image without fringe projection. (**c**) Original projection fringe pattern. (**d**) Optimized projection fringe pattern. (**e**) A captured fringe pattern during red light projection. (**f**) A captured fringe pattern during green light projection. (**g**) A captured fringe pattern during blue light projection. (**h**) Optimized captured fringe pattern using the method proposed in this paper. (**i**) Reconstructed 3-D surface shape during red light projection. (**j**) Reconstructed 3-D surface shape during green light projection. (**k**) Reconstructed 3-D surface shape during blue light projection. (**l**) Reconstructed 3-D surface shape using the method proposed in this paper.

Figure 10a shows an object with different colors on the surface. When the fringe pattern is not projected, the ambient light can be considered as white light, and the image captured by the camera is shown in Fig. 10b. It can be seen that different colors have different absorption and reflectance of white light, and the white surface has the highest reflectance. Figure 10c is the original projection fringe pattern. Figure 10d is the optimized projection fringe pattern obtained by the method proposed in this paper. The pixel grayscale and color are adjusted according to the reflectivity and color of the object surface. Figure 10e–g are the captured images during red light projection, green light projection and blue light projection. Figure 10h is the image captured based on the method proposed in this paper. It can be seen that Fig. 10h is clearer than Fig. 10e–g, especially the contrast and brightness of the non-white areas are better. Figure 10i–k shows the 3-D reconstructed shapes during red light projection, green light projection and blue light projection. 3-D reconstruction surface corresponding to the blue and green areas during red light projection has a large number of cavities or wrinkles. 3-D reconstruction surface corresponding to the blue and red areas during green light projection also has a large number of cavities or wrinkles. 3-D reconstruction surface corresponding to the green and red areas during blue light projection has a lot of cavities or wrinkles as well. Figure 10l is a 3-D reconstruction shape based on the method proposed in this paper. Its contour is complete and the contour wrinkles are significantly reduced.

**Figure 11:**
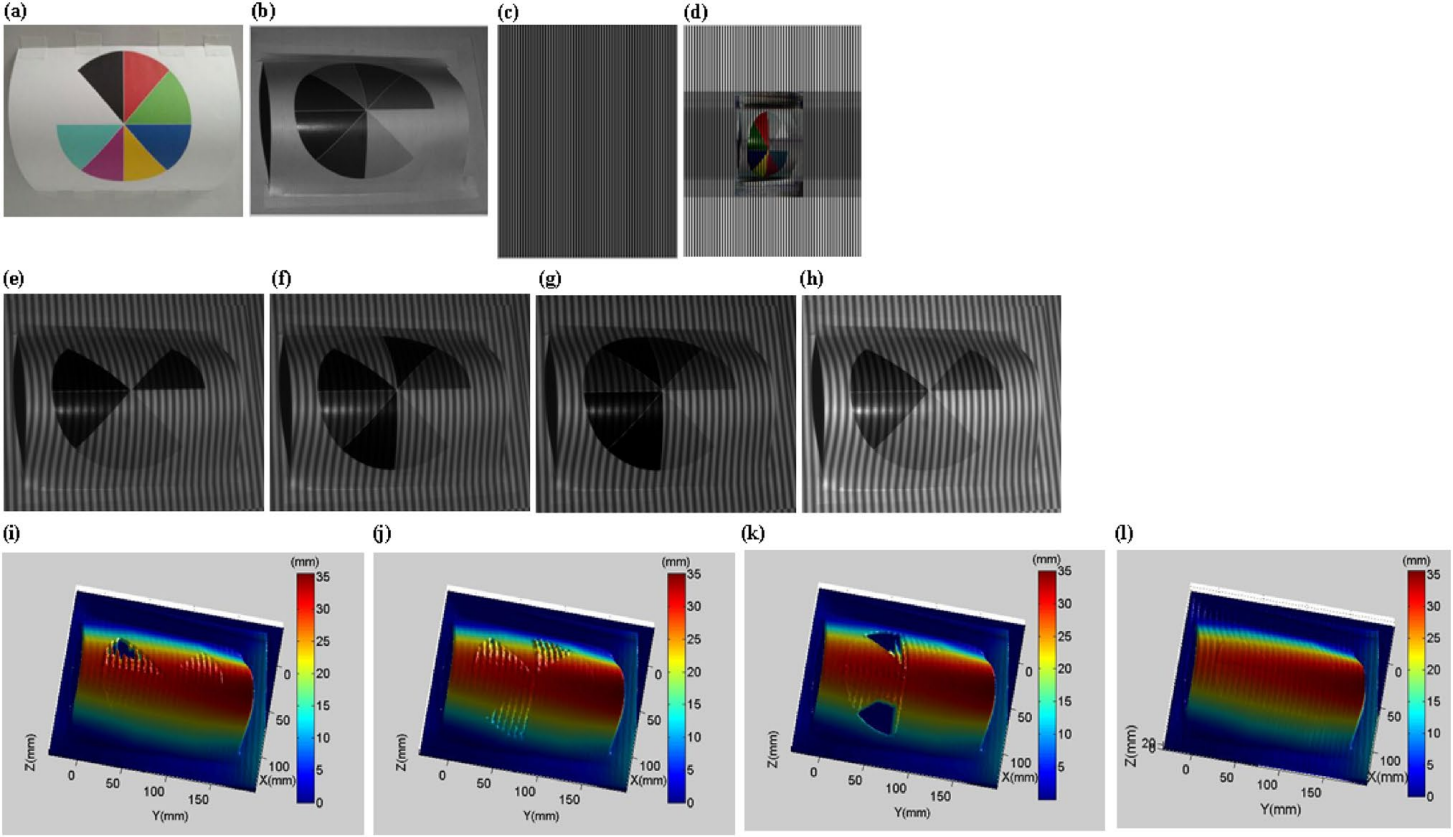
3-D shape reconstruction of a colorful object. (**a**) Another colorful object. (**b**) A captured image without fringe projection. (**c**) Original projection fringe pattern. (**d**) Optimized projection fringe pattern. (**e**) A captured fringe pattern during red light projection. (**f**) A captured fringe pattern during green light projection. (**g**) A captured fringe pattern during blue light projection. (**h**) Optimized captured fringe pattern using the method proposed in this paper. (**i**) Reconstructed 3-D surface shape during red light projection. (**j**) Reconstructed 3-D surface shape during green light projection. (**k**) Reconstructed 3-D surface shape during blue light projection. (**l**) Reconstructed 3-D surface shape using the method proposed in this paper.

In order to further verify the effectiveness of the proposed method, we use another colored object as the measured object. Through the comparison in Fig. 11, it is further verified the effectiveness of the proposed method.

The quantitative comparison results of RMSE are shown in Table 5. Compared with the RMSE of single red light, green light and blue light projection, the proposed method improves by 79.6%, 77.9% and 88.4%, respectively. Figure 10i–k all have cavities, and MDs are 26.53 mm, 34.47 mm and 48.71 mm, respectively. However, the MD of Fig. 10l is greatly reduced to 0.69 mm. Figure 11i,k also have cavities, and MDs are 25.58 mm and 30.45 mm, respectively. Although Fig. 11j does not have cavities, the wrinkles have a large amplitude and a wide range. The MD of Fig. 11l is reduced to 0.72 mm. It is worth noting that the nonlinear gamma of the projector causes wrinkles in the reconstructed 3-D shape, as shown in Figs. 10l and 11l. We will further study in the future work.

**Table 5:** Quantitative comparisons of colorful objects (RMSE).

Object	Red light projection	Green light projection	Blue light projection	Proposed method
Colorful object 1	0.324	0.298	0.571	0.066
Colorful object 2	0.255	0.234	0.450	0.052

## Conclusion

In this paper, we have proposed a novel high dynamic range 3-D shape measurement method based on adaptive adjustment of projection pixel intensity and camera exposure time. Firstly, according to the captured fringe pattern model, we have determined the optimal ratio of projected fringe intensity/background intensity to improve the SNR of the captured fringe pattern. Secondly, we set the camera exposure time to be smaller and project two images with a certain gray value respectively, which ensures that the captured image is not exposed. We determine the background intensity and the reflectivity of the object surface based on the above two captured images. Thirdly, according to the constraints of the fringe image intensity range and camera exposure time range, we propose a new algorithm to optimize the projection pixel intensity in the camera coordinate system and camera exposure time. Furthermore, we obtain the projection background intensity and modulation intensity according to the optimal ratio between them. Finally, we propose a novel method to map the pixels in the camera image coordinate system to the pixels in the projection image coordinate system. This process is fast due to the high-speed projection of binarized Gray coded fringe pattern.

This novel integrated method can obtain adaptive projection image and optimal exposure time, and is also very efficient in terms of efficiency, so it can be applied to high dynamic range 3-D shape measurement. There are two aspects that need to be further improved in the proposed method. (1) Due to the different shapes of various objects, one pixel mapping cannot be applied to different objects, although our method is highly efficient. In the future, this issue can be further considered to further improve measurement efficiency. (2) In the following work, we will consider omitting two image projections and using other methods to obtain the reflectivity of the measured objects and background intensity.
